# Supplementary material for: Self-probing spectroscopy of XUV photo-ionization dynamics in atoms subjected to a strong-field environment
Source: Nat Commun. 2017 Nov 13;8:1453. doi: 10.1038/s41467-017-01723-w (PMC5682292; doi:10.1038/s41467-017-01723-w)
Supplement: Supplementary file 1 — Supplementary Information [file 41467_2017_1723_MOESM1_ESM.pdf]

SUPPLEMENTARY FIGURES

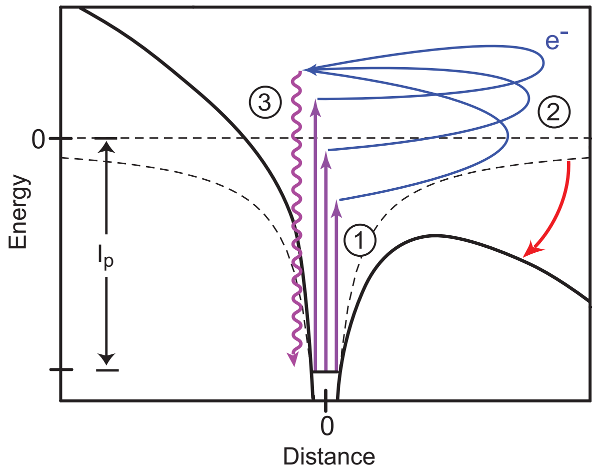

Supplementary Figure 1. **Illustration of the Coulomb-corrected three-step model.** Harmonics of the incoming extreme ultraviolet (XUV) attosecond pulse train ionize the atom (purple arrows); electrons are liberated by over-the-barrier emission over the combined laser-atom potential barrier (step 1). The resulting wavepacket is accelerated along different quantum paths (blue) and is driven back to the ionic core (step 2). In step 3, the quantum paths interfere with each other as recollision takes place, and recombination leads to the emission of a high-energy XUV photon.

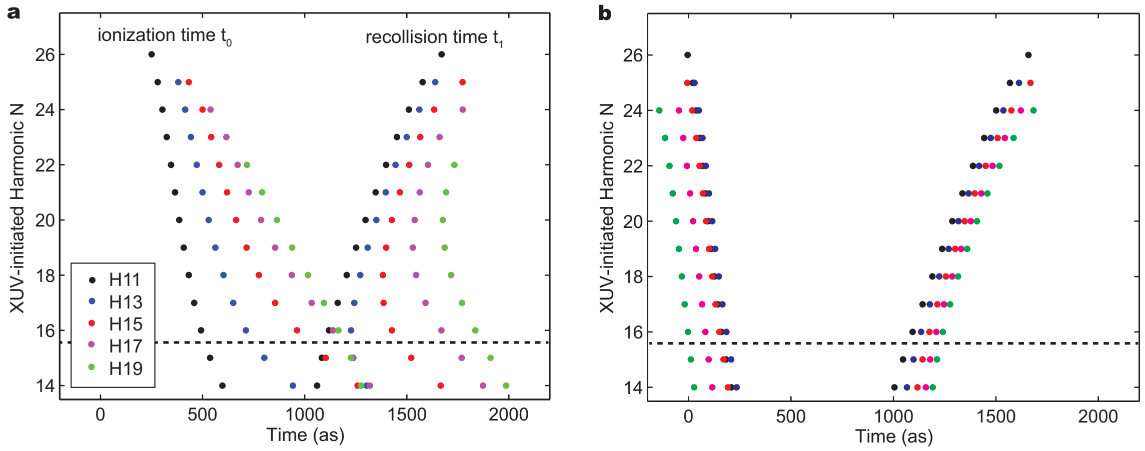

Supplementary Figure 2. **Ionization and recombination times.** **a**, t_0 (earlier times) and t_1 (later times) for downhill trajectories for different input harmonics n (see legend). **b**, Uphill trajectories. The dashed line indicates the helium ionization threshold.

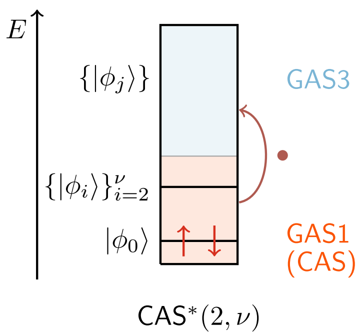

Supplementary Figure 3. **GAS division used in this work.** Orbitals (labeled by $|\phi_i\rangle$) of different spin are assumed to have the same energy. The (red) arrow shows the allowed single excitations. The GAS[1] describes a complete-active space with ν spatial orbitals and excitations out of the CAS (CAS$^*(2,\nu)$).

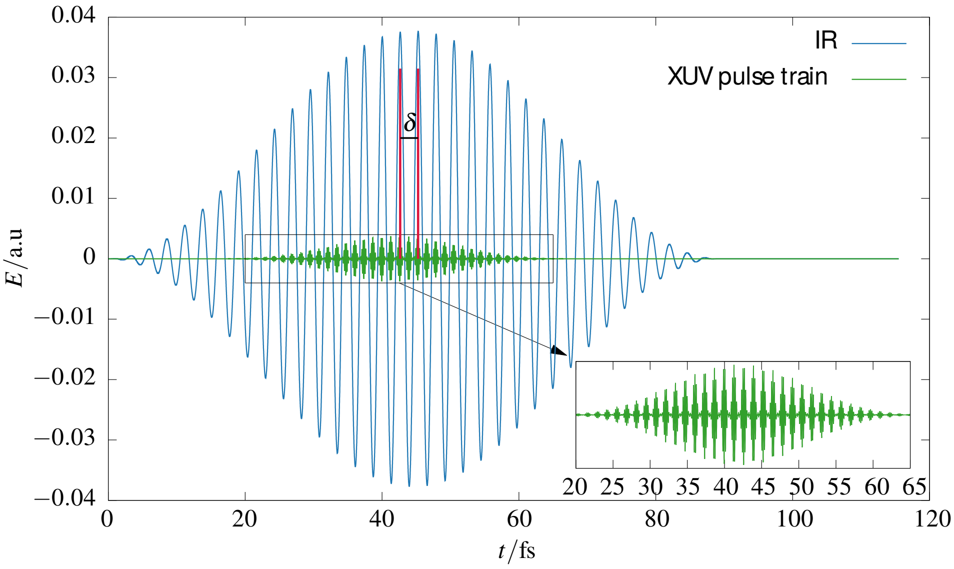

Supplementary Figure 4. **Example of electric fields used in the simulation.** The blue (green) curve shows the IR pulse (APT). The red lines indicate the offset δ between the IR and the APT, which is one IR cycle in this example. Here the IR field strength is 0.0377 a.u. (5×10^{13} W cm^{-2}).

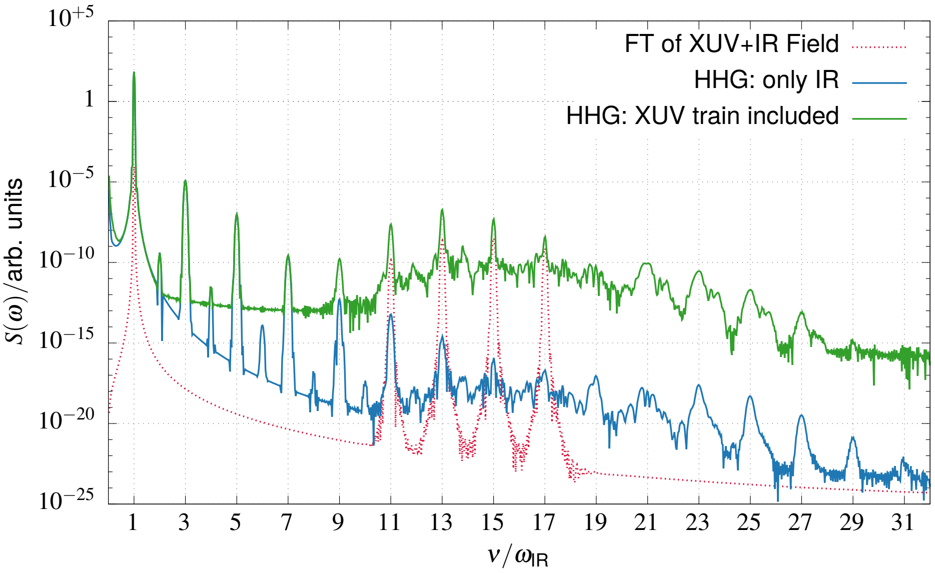

Supplementary Figure 5. **Simulated HHG spectra.** At zero delay ($\delta = 0$) for an IR field strength of 0.0377 a.u. (5×10^{13} W cm^{-2}). Shown is the spectrum for the simulation with only the IR pulse (blue) and for the simulation with the IR pulse and the APT (green). For clarification, the Fourier transform of the field of the IR pulse and the APT is shown as well (red). Note the logarithmic scale of the spectrum.

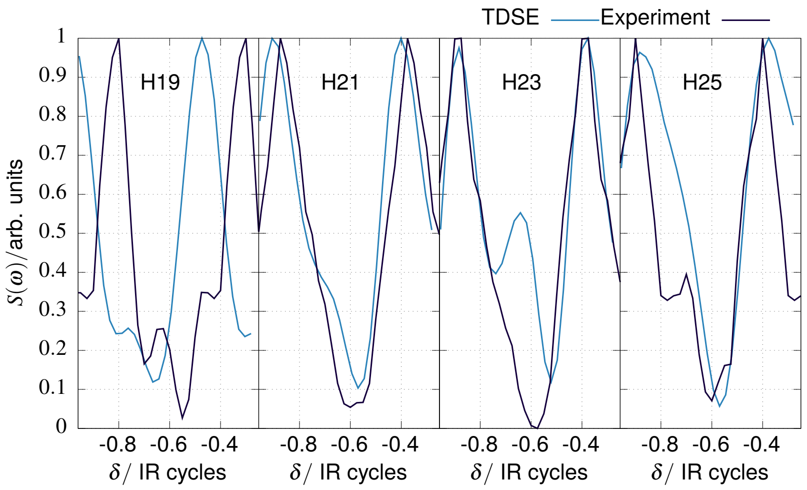

Supplementary Figure 6. **Simulated XUV-IR delay scan results.** We show the results of the TDSE simulations (blue) and the experimental data (black) for harmonics 19 to 25. The IR field strength is 0.0477 a.u. (8×10^{13} W cm^{-2}).

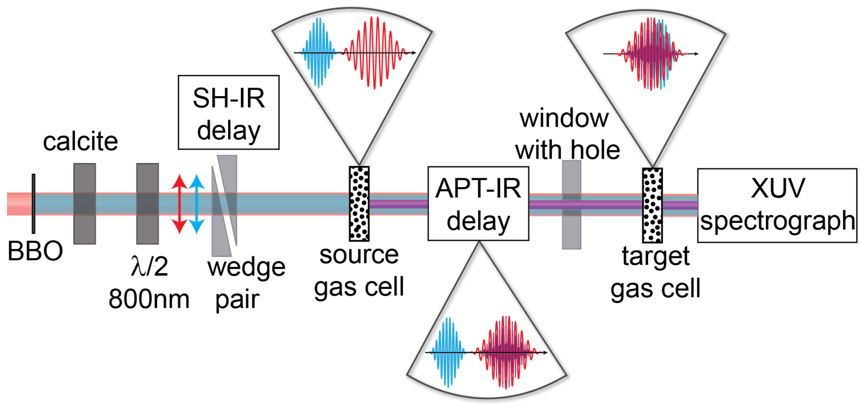

Supplementary Figure 7. **Experimental set-up for perturbative measurements.** Before the IR beam (red lines) enters the source gas cell, it is frequency doubled by a thin BBO crystal (blue lines). Calcite windows in conjunction with a fused silica window after the source gas cell control the place in which the SH and IR pulses are synchronized (synchronization in the target gas cell is exemplified here). The fused silica window has a central hole in order to allow transmission of the APT (purple lines). Following the calcite windows, a half wavelength waveplate is used for rotating the IR polarization to match the SH polarization direction. The sub-cycle SH-IR delay is controlled by a fused silica wedge pair. The XUV-IR delay control and the spectrum measurement are the same as in the one-dimensional measurement.

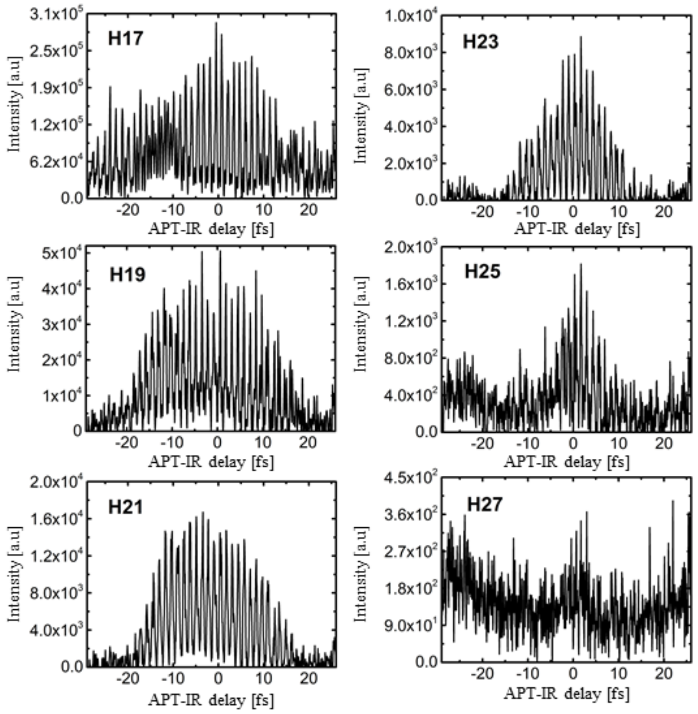

Supplementary Figure 8. **Long range delay scan with helium in the target gas cell.** Intensity of harmonics 17 to 27 as function of the relative XUV-IR delay. Zero delay is defined arbitrarily.

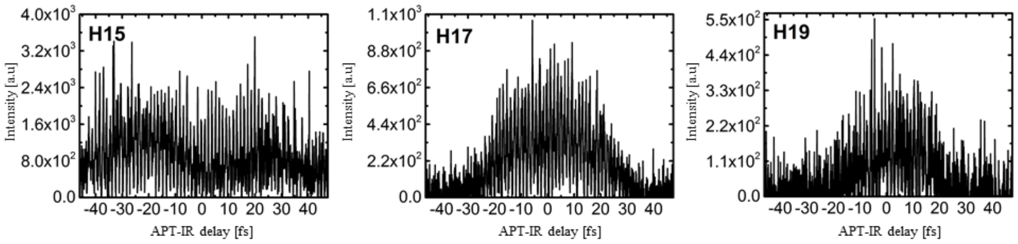

Supplementary Figure 9. **Long range delay scan with neon in the target gas cell.** Intensity of harmonics 17 to 27 as function of the relative XUV-IR delay. Zero delay is defined arbitrarily.

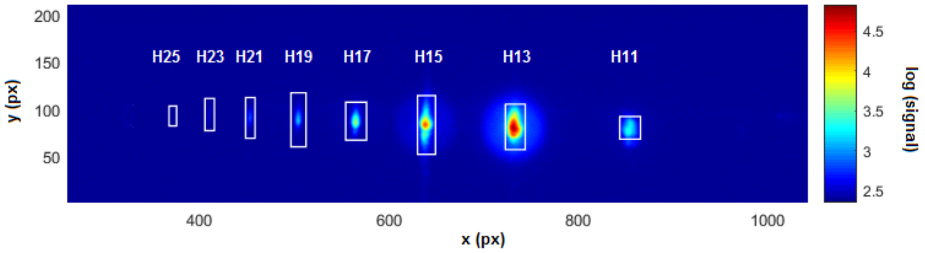

Supplementary Figure 10. **Example spectrum.** Image of the MCP detector screen with the CCD camera. The XUV-IR delay is set so that the intensity of the new harmonics is maximized. All harmonics and the corresponding regions of interest are denoted by white boxes.

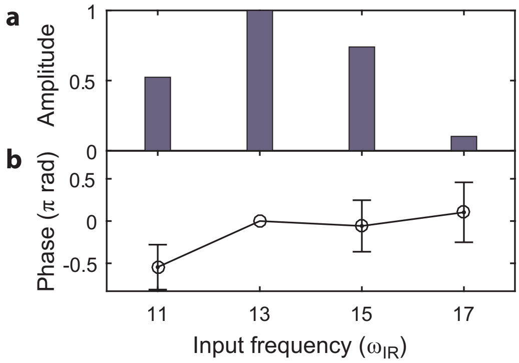

Supplementary Figure 11. **Spectral properties of the APT.** Relative amplitudes (a) and relative phases (b) of the input harmonics, with H13 as reference. The error bars indicate the standard deviation.

SUPPLEMENTARY TABLES

Description	Definition
Phase of the XUV components	$\beta_n \in \{0, \pi, 0, \pi\}$
Weights of the XUV components	$b_n \in \{0.2214, 0.4227, 0.3127, 0.0432\}$
Frequency of the IR field	$\omega = 0.0579 \quad (787\,\mathrm{nm})$
Amplitude of the IR field	$E_0^{\mathrm{IR}} = 0.0477 \quad (8 \times 10^{13}\,\mathrm{W\,cm^{-2}})$
Amplitude of the XUV field	$E_0^{\mathrm{XUV}} = 0.00119 \quad (5 \times 10^{10}\,\mathrm{W\,cm^{-2}})$
Duration of the IR field	$T^{\mathrm{IR}} = 3.6896 \times 10^3 \quad$ (32.49 fs FWHM in intensity)
Duration of the XUV field	$T^{\mathrm{XUV}} = 2.3931 \times 10^3 \quad$ (15.12 fs FWHM in intensity)

Supplementary Table 1. **Time-dependent Schrödinger equation simulation parameters.** Parameters for the fields corresponding to Supplementary Equations 26, 27, 28

SUPPLEMENTARY NOTES

Supplementary Note 1: Coulomb-corrected three-step model

In this section, we detail the Coulomb-corrected three-step model (CCTSM), a simple model for XUV-initiated high-harmonic generation (HHG). It is motivated by the fact that the semi-classical three-step model[2] needs to be extended in order to describe photoionization by an XUV attosecond pulse train (APT) and the resulting XUV-IR delay oscillations.

The CCTSM describes XUV-initiated HHG in three steps (see Supplementary Figure 1 for an illustration): Photoionization by the XUV field via over-the-barrier emission (OBE), propagation of the electron in the combined laser-Coulomb potential and recombination into the ground state. Starting from a strong-field approximation approach[3–5], we generalize the model to include OBE and the influence of the atomic potential[6]. OBE essentially enables photoionization by XUV absorption and subsequent electron emission over the potential barrier lowered by the IR field. In addition, the excited states under the influence of the strong IR field are treated as a quasi-continuum, motivated by theoretical predictions[7,8]. These modifications are essential since we experimentally observe a strong contribution of the full spectral bandwidth of the APT to the photo-ionization – although H11 to H15 are not able to directly ionize helium by themselves when ignoring the effects of the IR field and the Coulomb potential. Although we simplify the interplay of XUV (driving transitions to a quasi-continuum) and IR (creation of a potential barrier that can be passed by an electron without tunneling), the model captures the essential features of the experiment.

Lewenstein-like ansatz to the problem

We begin our derivation of the model based on the Lewenstein's seminal paper[3], closely following the work of Leeuwenburgh and co-authors[5]. Throughout this section we use atomic units (a.u.). The IR field $\mathbf{E}_{\mathrm{IR}}(t)$ and the XUV field $\mathbf{E}_{\mathrm{XUV}}(t)$ are defined as

$$\mathbf{E}_{\mathrm{IR}}(t) = \mathbf{e}_z E_{\mathrm{IR}}(t) = \mathbf{e}_z E_0^{\mathrm{IR}} \cos(\omega t), \tag{1}$$

$$\mathbf{E}_{\mathrm{XUV}}(t) = \mathbf{e}_z E_{\mathrm{XUV}}(t) = \mathbf{e}_z E_0^{\mathrm{XUV}} \sum_n b_n \cos(n\omega t - \beta_n). \tag{2}$$

Note that the XUV APT is decomposed into a Fourier series with individual weights, b_n, and phases, β_n. Both fields are linearly polarized along axis z (unit vector $\mathbf{e}_z$). The IR vector potential is defined as $\mathbf{A}_{\mathrm{IR}}(t) = -\mathbf{e}_z E_{\mathrm{IR}}/\omega \sin \omega t$.

Next, we formulate the time-dependent dipole along the laser polarization axis

$$d(t) = i \int_{-\infty}^{t} dt' \int d^3\mathbf{p}\, E_{\mathrm{XUV}}(t')\, d_z(\mathbf{p} + \mathbf{A}_{\mathrm{IR}}(t'))\, \exp[iS(\mathbf{p}, t, t')]\, d_z^{\star}(\mathbf{p} + \mathbf{A}_{\mathrm{IR}}(t)) + c.c. \tag{3}$$

First, we assume that ionization is dominated by the APT; hence, we only consider transitions from the ground state to Volkov states, mediated by the rapidly oscillating XUV electric field $\mathbf{E}_{\mathrm{XUV}}(t)$. Here we define $d_z(\mathbf{v})$ as the projection of the dipole matrix element connecting the ground state to a Volkov state $\mathbf{v}$ onto the polarization axis. Hence the term $E_{\mathrm{XUV}}(t')\, d_z(\mathbf{p} + \mathbf{A}_{\mathrm{IR}}(t'))$ describes the photoionization step. Second, we assume that the propagation of the ionized electron wavepacket is dominated by the slowly varying IR field. The quasi-classical action S describes the phase evolution of the photoelectron and the ground state and is given by

$$S(\mathbf{p},t,t') = -\int_{t'}^{t} dt'' \left(\frac{[\mathbf{p}+\mathbf{A}_{\mathrm{IR}}(t'')]^2}{2} + I_{\mathrm{p}} \right). \tag{4}$$

Last, the term $d_z^\star(\mathbf{p}+\mathbf{A}_{\mathrm{IR}}(t))$ in Supplementary Equation 3 accounts for the recombination step.

We now apply the saddle point approximation in the canonical momentum $\mathbf{p}$ and find

$$d(t) = i \int_{-\infty}^{t} dt' \left(\frac{\pi}{\eta + \frac{i}{2}(t-t')} \right)^{3/2} E_{\mathrm{XUV}}(t')\, d_z(\mathbf{p}_{\mathrm{st}}+\mathbf{A}_{\mathrm{IR}}(t')) \\ \times \exp[iS(\mathbf{p}_{\mathrm{st}},t,t')]\, d_z^\star(\mathbf{p}_{\mathrm{st}}+\mathbf{A}_{\mathrm{IR}}(t)) + c.c., \tag{5}$$

with $\eta \ll 1$ and the saddle point momentum

$$\mathbf{p}_{\mathrm{st}}(t,t') = -\frac{1}{t-t'} \int_{t'}^{t} dt'' \mathbf{A}_{\mathrm{IR}}(t''). \tag{6}$$

Note that the first term of Supplementary Equation 5 in large brackets is identified with the spreading of the free electron wavepacket during propagation, decreasing the overlap with the ground state with increasing time. The harmonic spectrum follows from $I(N\omega) \propto (N\omega)^4 \, |d(N\omega)|^2$, with the frequency dipole

$$d(N\omega) = i \int_{-\infty}^{+\infty} dt \exp(iN\omega t) \int_{-\infty}^{t} dt' \left(\frac{\pi}{\eta + \frac{i}{2}(t-t')} \right)^{3/2} E_{\mathrm{XUV}}(t')\, d_z(\mathbf{p}_{\mathrm{st}}+\mathbf{A}_{\mathrm{IR}}(t')) \\ \times \exp[iS(\mathbf{p}_{\mathrm{st}},t,t')]\, d_z^\star(\mathbf{p}_{\mathrm{st}}+\mathbf{A}_{\mathrm{IR}}(t)) + c.c., \tag{7}$$

where N can be identified with the harmonic order of the emitted XUV light.

Saddle point approximation for XUV-initiated HHG

The natural interpretation of t' and t (Supplementary Equation 7) are ionization time and recollision time, respectively. Carrying out a saddle point approximation with t and t' naturally leads to the notion of electron trajectories. Owing to the high-frequency XUV field, a saddle point approach for t' cannot be applied for S as defined earlier, unlike tunneling-initiated HHG. However, we can include the rapidly oscillating XUV field in S and hence circumvent this issue. A similar approach was chosen independently in another work[4]. After completing the following derivation, we will discuss the absence of IR-induced tunneling in XUV-initiated HHG as evidenced by the experiment.

For simplicity we will focus only on a single ionizing XUV frequency $n\omega$. The term in the exponent for the saddle point approach with the rotating component $\exp(-in\omega t')$ of the XUV field then becomes:

$$\Theta_{\mathrm{XUV}-}(\mathbf{p}_{\mathrm{st}},t,t') = -n\omega t' - \int_{t'}^{t} dt'' \left(\frac{[\mathbf{p}_{\mathrm{st}}(t,t'')+\mathbf{A}_{\mathrm{IR}}(t''))]^2}{2} + I_{\mathrm{p}} \right) + N\omega t. \tag{8}$$

Together with the saddle point equation for $\mathbf{p}_{\mathrm{st}}$, we retrieve a set of three equations:

$$\frac{\partial \Theta_{\mathrm{XUV}-}(\mathbf{p},t,t')}{\partial t'}|_{\mathbf{p}_{\mathrm{st}},t_1,t_0} \rightarrow \frac{1}{2}\left[\mathbf{p}_{\mathrm{st}}+\mathbf{A}_{\mathrm{IR}}(t_0)\right]^2 = -I_{\mathrm{p}} + n\omega, \tag{9}$$

$$\frac{\partial \Theta_{\mathrm{XUV}-}(\mathbf{p},t,t')}{\partial \mathbf{p}}|_{\mathbf{p}_{\mathrm{st}},t_1,t_0} \rightarrow \int_{t_0}^{t_1} dt'' \left[\mathbf{p}_{\mathrm{st}}+\mathbf{A}_{\mathrm{IR}}(t'')\right] = 0, \tag{10}$$

$$\frac{\partial \Theta_{\mathrm{XUV}-}(\mathbf{p},t,t')}{\partial t}|_{\mathbf{p}_{\mathrm{st}},t_1,t_0} \rightarrow \frac{1}{2}\left[\mathbf{p}_{\mathrm{st}}+\mathbf{A}_{\mathrm{IR}}(t_1)\right]^2 = -I_{\mathrm{p}} + N\omega, \tag{11}$$

where t_0 and t_1 are the obtained saddle times from t' and t, respectively. We also obtain a solution $\Theta_{\mathrm{XUV}+}(\mathbf{p}, t, t')$ that includes $\exp(+in\omega t')$ of the XUV field. This counterrotating solution does not play any role because of the extremely large imaginary part of the corresponding saddle value t_0.

Transversal components of p do not contribute to the dipole along the laser polarization axis $\mathbf{e}_z$, so we confine it to one dimension ($\mathbf{p} \to p$, etc.). The resulting saddle point equations give us a natural insight into the physical mechanism: First, we ionize the atom by XUV photoionization, then the electron is driven back to the ionic core, and it finally recombines, leading to the emission of a high-energy photon. The ionization step is mediated by an XUV photon, which results in a modified initial energy $n\omega - I_\mathrm{p}$. If the energy of the incoming XUV photon is larger than the ionization energy I_p, we retrieve a real-valued saddle time t_0. The solution naturally provides us with the excess energy of the liberated electron. Since linear single-photon ionization shows no directionality in electron velocity, we obtain two branches of solutions for t_0 (and also for the other saddle variables), denoted as $t_{0(+)}$ and $t_{0(-)}$:

$$p_\mathrm{st} + A_\mathrm{IR}(t_{0(\pm)}) = \pm\sqrt{2\,(-I_\mathrm{p} + n\omega)}. \tag{12}$$

This means that the XUV photon energy has a strong influence on the timings of the electron trajectory via the excess kinetic energy of the photoelectron. The two classes (+) and (−) can be identified with downhill (extra kick away from the nucleus) or uphill (extra kick towards the nucleus), respectively, in line with earlier findings[6,9–11]. If the energy of the XUV photon cannot liberate the electron, then the first step of the HHG process is brought about by a combination of XUV excitation and IR tunneling ionization. Here the saddle times and the momentum acquire imaginary components.

Finally, we obtain the following expression for the frequency dipole:

$$\begin{aligned} d(N\omega) = \frac{1}{2}\sum_{j=1}^{M} \frac{2\pi}{\sqrt{\det \mathcal{H}\,\Theta_{\mathrm{XUV}-}(t,t')|_{t_1^{(j)},t_0^{(j)}}}} \left(\frac{\pi}{\eta + \frac{i}{2}(t_1^{(j)} - t_0^{(j)})}\right)^{3/2} E_0^\mathrm{XUV}\, b_n \exp\left(-i\beta_n\right) \\ \times\, d_z(p_\mathrm{st}^{(j)} + A_\mathrm{IR}(t_0^{(j)}))\, \exp[i\Theta_{\mathrm{XUV}-}(p_\mathrm{st}^{(j)}, t_1^{(j)}, t_0^{(j)})]\, d_z^\star(p_\mathrm{st}^{(j)} + A_\mathrm{IR}(t_1^{(j)})), \end{aligned} \tag{13}$$

where M indicates the number of trajectories within a full cycle of the IR and $\mathcal{H}$ indicates the Hessian of the function that follows it.

In the experiment, the majority of input harmonics can't directly ionize the helium atom without the assistance of the IR field. However, the experimental results clearly show that all input harmonics lead to photoionization. Hence, within the framework of the present model, IR-induced tunneling ionization from a transiently excited state must necessarily take place, leading to imaginary saddle times and imaginary S. However, depending on the magnitude of the effective ionization potential given by the difference of the XUV photon energy and the binding energy, these imaginary components will induce a strong dampening of the resulting dipole. If we take multiple input harmonics into consideration, this will also result in dampening of the XUV-IR oscillations. In the experiment, however, we do not observe strong dampening; the contrast of the oscillations is very high (see Supplementary Figure 8). Thus, we conclude that the contribution of IR-induced tunneling ionization is likely to be very small or even non-existent. This is confirmed by the second harmonic perturbative experiments (see Figure 3 in the main text). An alternative approach to describe the interplay between XUV and IR is provided by OBE. In the following section we will outline the necessary modifications to the theoretical model.

Over-the-barrier emission in the combined laser-atom-potential

Here we consider OBE, closely following Ref. 6 and modifying the model that we formulated in the last paragraph. OBE is an alternative way to describe the interplay of IR and XUV in the process. Here electrons undergo transitions from the ground state to transiently excited states. There they possess sufficiently large kinetic energy to overcome the potential barrier created by the IR field and the Coulomb potential, hence they can be considered as ionized. Note that throughout the main text and from this point in this Supplementary Note we employ the term XUV photoionization although the ionization process technically is a combination of XUV photoexcitation and subsequent OBE.

Because the electrons are propagating in the combined laser-atom potential, the canonical momentum p is no longer well-defined; recollision trajectories and the associated ionization and recombination times have to be determined numerically. We calculate short electron trajectories in the combined laser-atom potential by numerically integrating Newton's equations of motion. Here we use a 4th order Runge-Kutta-Fehlberg algorithm[12], considering a 1D softcore-Coulomb potential of the form

$$V(z) = -\frac{Z_{\text{eff}}}{\sqrt{a^2 + z^2}}, \tag{14}$$

representing helium with effective nuclear charge $Z_{\text{eff}} = 1.353$ and softening parameter $a^2 = 0.001$[13]. For a set of ionization times t_0 we launch electrons at $z_0 = 0$ with initial velocity $v_0 = \pm\sqrt{2(-I_{\text{p}} - V(z_0) + n\omega)}$, corresponding to the kinetic energy of a photoionized electron within the atomic potential. For those electrons subjected to OBE we also have to consider both downhill (+) and uphill (−) trajectories. We propagate electrons of both trajectory classes and calculate the first return time $t_{1(+)}$ for downhill trajectories and the second return time, denoted as $t_{1(-)}$, for uphill trajectories. For each trajectory we determine the corresponding final kinetic energy and the accumulated classical action $S_{\text{OBE,cl}}(t, t')$. We register final kinetic energies corresponding to the emission of a harmonic photon (energy $N\omega$) and thus obtain a full solution with emission time, recombination time and action, linking an input harmonic n to an output harmonic N.

Having obtained OBE trajectory solutions, we now make use of Supplementary Equation 13 which was derived in the previous subsection, but in a strongly reduced form. For simplicity we retain only the essential term that describes the broadening of the electron wavepacket during (Coulomb-free) propagation[14] and the exponential term containing the (numerically calculated) action, the evolution of the ground state and the frequency of the absorbed and emitted photons. A strong modification, however, is neglecting the momentum dependence of the dipole matrix elements. Therefore we make no distinction between ionization to continuum states and excitation to states below the ionization threshold – everything is treated effectively as ionization to a continuum. At IR intensities similar to those applied in our experiment ($5...10 \times 10^{13}\,\text{W}\,\text{cm}^{-2}$), bound-state resonances in singly-excited helium are strongly shifted and broadened by the laser field[7,8]. In the experiment, we do not observe signatures of pronounced excited state or continuum resonances. A full treatment of these resonances including transient absorption phenomena can be performed with extensive *ab-initio* modeling, but a conclusive measurement, for example with attosecond transient absorption, remains elusive to date to the best of our knowledge. For our purposes a simple model based on the aforementioned approximations was sufficient. The frequency dipole now reads,

$$d(N\omega) \propto \sum_{j=1}^{M} a_n \exp\left(-i\phi_n\right) \left(\frac{\pi}{\eta + \frac{i}{2}(t_1^{(j)} - t_0^{(j)})}\right)^{3/2} \exp[i\Theta_{\text{XUV-,OBE}}(t_1^{(j)}, t_0^{(j)})], \tag{15}$$

with the redefined term in the exponent

$$\Theta_{\mathrm{XUV-,OBE}}(t,t') = -n\omega t' - S_{\mathrm{OBE,cl}}(t,t') - I_{\mathrm{p}}(t-t') + N\omega t. \tag{16}$$

Here we integrated the complex dipole matrix element for XUV photoionization and the XUV's amplitude and phase into new parameters a_n and ϕ_n which form together the complex ionization amplitude

$$a_n \exp(-i\phi_n) = b_n \exp(-i\beta_n) d_z(\mathbf{p}_{\mathrm{st}} + \mathbf{A}_{\mathrm{IR}}(t')). \tag{17}$$

a_n and ϕ_n effectively describe the XUV photoionization process and ultimately the build-up of the XUV-initiated electron wavepacket. As it can be seen from Supplementary Equation 16, we recover the intuitive three-step picture of photon absorption ($-n\omega t'$), electron propagation ($S_{\mathrm{OBE,cl}}(t,t')$) and ground state evolution ($-I_{\mathrm{p}}(t-t')$), and finally photon emission ($+N\omega t$). No tunneling is involved; the electron trajectories are treated in a completely classical way.

Dependence of XUV-initiated HHG on XUV-IR delay

We will now examine the case of multiple XUV frequencies where we sum over their respective contributions to the final spectrum. This is required for XUV-IR delay dependence within our model. With two neighboring harmonics of one kind (even or odd), the final dipole frequency spectrum $|d(N\omega)|^2$ is a result of XUV-IR delay dependent quantum interference of the respective trajectory pair. For the sake of illustration we assume input harmonics n and $n+2$ with equal excitation amplitudes $a_n = a_{n+2}$ and equal prefactor $f = \pi / \left[\eta + i/2 \left(t_1^{(n)} - t_0^{(n)}\right)^{3/2}\right]$ and calculate the intensity of an XUV-initiated harmonic N:

$$|d(N\omega)|^2 = C|fa_n|^2 \Bigg| \exp\left\{-n\omega(t_0^{(n)} - \Delta t) - S_{\mathrm{OBE,cl}}(t_1^{(n)}, t_0^{(n)}) - I_{\mathrm{p}}(t_1^{(n)} - t_0^{(n)}) + N\omega t_1^{(n)}\right\} + \exp\left\{-(n+2)\omega(t_0^{(n+2)} - \Delta t) - S_{\mathrm{OBE,cl}}(t_1^{(n+2)}, t_0^{(n+2)}) - I_{\mathrm{p}}(t_1^{(n+2)} - t_0^{(n+2)}) + N\omega t_1^{(n+2)}\right\} \Bigg|^2 \tag{18}$$

We see that the interference term is proportional to $\cos(\Delta n \omega \Delta t - \varphi_{\mathrm{osc}}(N))$, where $\Delta n = 2$ is the difference in harmonic number, Δt is the XUV-IR delay and $\varphi_{\mathrm{osc}}(N)$ is the oscillation phase that mainly originates from the difference in ionization times. This introduces a simple half-cycle delay dependence into the model. The phase $\varphi_{\mathrm{osc}}(N)$ and the corresponding optimal XUV-IR delay are related, but not identical, to the average ionization time of the trajectory pair. A Fourier analysis clearly shows an oscillatory behavior with the main component oscillating at twice the IR frequency, but also weaker oscillations at 4ω and 6ω (see Figure 2 in the main text). These higher frequency components arise from the interference of trajectories from harmonics with $\Delta n = 4$ and $\Delta n = 6$, respectively. This defines the multiple path quantum interferometer, the main focus of the of this work. Even higher frequencies can result from interference of an ionizing harmonic with an XUV-initiated harmonic of the same frequency[15]. However, this type of oscillation was not observed in the experiment since most XUV-initiated high harmonics are background-free; the only exception is H19.

Conservation of parity

In the following we will evaluate how parity is transferred from the input harmonics to the XUV-initiated harmonics. We assume that ionization is triggered either by an even or an odd harmonic n. We consider two OBE trajectories in subsequent half cycles (A) and (B), all corresponding to a trajectory leading to the emission of harmonic N – one trajectory being the exact mirror image of the other. Evaluating $|d(N\omega)|^2$ then yields

$$|d(N\omega)|^2 \propto \cos^2\left[(n-N)\pi/2 + (S^{(A)}_{\mathrm{OBE,cl}} - S^{(B)}_{\mathrm{OBE,cl}})/2\right], \tag{19}$$

with $S^{(A)}_{\mathrm{OBE,cl}}$ and $S^{(B)}_{\mathrm{OBE,cl}}$ being the corresponding action terms. Due to the symmetry of the trajectories, the action terms are equal and cancel each other. It is obvious that only for even $(n-N)$ constructive interference is obtained. Therefore, the parity of the harmonics is conserved in the process. This is in line with the experimental observation that the parity of the input harmonics is transferred directly to the output harmonics (see Figure 3d and e in the main text). Hence the effects of even and odd input harmonics can be treated separately in the model.

Calculation details

In order to apply the model to describe the experiment, we will consider short trajectories only. We take into account both downhill and uphill trajectories, with the former naturally dominating over the latter due to shorter excursion times. We target the helium atom ($I_\mathrm{p} = 24.59\,\mathrm{eV}$), the IR intensity is $8 \times 10^{13}\,\mathrm{W\,cm^{-2}}$, and the wavelength is 787 nm for the strong-field model calculation used in the reconstruction of a_n and ϕ_n (see Figure 4 in the main manuscript).

First, we describe trajectory calculations for a set of odd input harmonics n ranging from H11 to H19. Supplementary Figure 2 displays ionization times t_0 and recollision times t_1 of output harmonics H14 to H26 for downhill and uphill electrons, respectively. The harmonic cut-off averaged over all input harmonics is in agreement with the simple three-step picture[2], $N_{\mathrm{max}}\omega \sim I_\mathrm{p} + 3U_\mathrm{p}$. Also the cut-off observed in the experiment agrees with the three-step picture. Note that in our specific experiment the downhill trajectory initiated by H13 dominates over all other channels. It is this trajectory that mainly defines the time-energy mapping of XUV-initiated HHG.

In the following, we will consider the experiment with the second harmonic (SH) of the IR present in the target gas cell (see Figure 3a-c, main text). The symmetry breaking between initially left- and right-moving trajectories by the SH field cannot be described in the simple phase perturbation picture (Eq. 2, main text), since the canonical momentum p is not well-defined for propagation in the Coulomb potential. Instead, we include the SH field directly into the trajectory calculations and repeat the simulation for a set of SH-IR phases. A 2D scan yields the SH-IR oscillation phases shown in Figure 3c in the main text after integration over XUV-IR delay. Here ε was taken as 0.05 and the IR intensity as $7.5 \times 10^{13}\,\mathrm{W\,cm^{-2}}$. We use the ionization amplitudes $a_{11} = 0.2055$, $a_{13} = 0.3584$, $a_{15} = 0.3831$ and $a_{17} = 0.0530$. We determine the ionization amplitudes by measuring the intensity difference of each harmonic when the APT and the IR pulse overlap in time compared to the case when helium is absent in the target gas cell. Furthermore, we assume a flat phase because the choice of the phase does not affect the response of XUV-initiated HHG to the SH field once we integrate over XUV-IR delay.

For simulating the XUV-initiated HHG with a pulse train consisting of even and odd harmonics (Figure 3d and e, main text), we extracted the SH-IR-phase-dependent ionization amplitudes $a_n(\varphi)$ from the measurements of the xenon harmonics. The coefficients

are defined as $a_n(\varphi) = \bar{a}_n + c_n \cos 2(\varphi - \varphi_{n,0})$, where $\bar{a}_n$ is the phase-averaged (DC) amplitude corresponding to the harmonic n, c_n the (AC) amplitude of the oscillation and $\varphi_{n,0}$ the optimal SH-IR phase as extracted from the experiment. We calculated a full XUV-IR delay scan for each SH-IR phase φ and the corresponding input spectrum, yielding the results shown in Figure 3e in the main text after integration over XUV-IR delay. The IR intensity was taken as $7.5 \times 10^{13}\,\mathrm{W\,cm^{-2}}$.

Supplementary Note 2: Integration of the time-dependent Schrödinger equation

In the following section, we will detail our calculations using the time-dependent Schrödinger equation (TDSE) that includes two-electron correlations.

Theory

For computing the HHG process, we focus on the non-relativistic, quantum-mechanical theory of a single helium atom, thereby neglecting macroscopic phase effects[16]. The dynamics are governed by the TDSE for the two-electron wave function. In atomic units ($m_e = e = 4\pi\epsilon_0 = 2|E_{\mathrm{Ryd}}| = a_0 = 1$), it reads as

$$\mathrm{i}\frac{\partial}{\partial t}\left|\Psi(t)\right\rangle = \hat{H}(t)\left|\Psi(t)\right\rangle, \tag{20}$$

with the time-dependent Hamiltonian in spatial representation

$$\hat{H}(t) = \sum_{i=1}^{2}\hat{h}_i(t) + \frac{1}{|\mathbf{r_1} - \mathbf{r}_2|}, \tag{21}$$

where $\mathbf{r}_i$ is the position operator of electron i. The single-particle Hamiltonian $\hat{h}_i(t)$ is

$$\hat{h}_i(t) = -\frac{1}{2}\nabla_i^2 - \frac{2}{|\mathbf{r}_i - \mathbf{R}|} + W(\mathbf{r}, t), \tag{22}$$

where $\mathbf{R}$ denotes the position operator of the nucleus. In the calculation, $\mathbf{R} = \mathbf{0}$ is fixed. The interaction with the field, $W(\mathbf{r}, t)$, is treated within the dipole approximation using the velocity gauge,

$$W(\mathbf{r}, t) = -\mathrm{i}\mathbf{A}(t) \cdot \nabla. \tag{23}$$

Here, $\mathbf{A}(t)$ is the vector potential of the corresponding electric field which is assumed to be linearly polarized in z direction. In order to exploit symmetry, Supplementary Equation (20) is transformed to spherical coordinates[17–19].

The HHG spectrum is computed by the Fourier transform of the dipole acceleration in the z direction, $\ddot{z}$,[19,20]

$$S^z(\omega) \propto \omega^{-4}\left|\int_0^{t_{\mathrm{final}}} \mathrm{d}t\,\langle\Psi(t)\,|\ddot{z}|\,\Psi(t)\rangle \exp(-\mathrm{i}\omega t)\right|^2. \tag{24}$$

Owing to the numerical complexity of the two-electron ionization dynamics, e.g., the HHG process, we approximate Supplementary Equation (20) using the time-dependent generalized-active-space configuration-interaction (TD-GAS-CI) framework[21–24]. Its accuracy has recently been demonstrated by simulating ionization dynamics in diatomic molecules[1]. In the TD-GAS-CI approximation, the many-particle wave function is expanded into a selection of time-independent Slater determinants $|\Phi_I\rangle$ via the GAS concept. This allows for an approximation based on physical assumptions, namely, that

double ionization is ignored. The Slater determinants themselves are antisymmetrized linear combinations of single-particle-functions (orbitals), following a partition-in-space-concept[22,23]. Thereby, an inner region in the vicinity of the nucleus and an outer region is defined. The outer region consists of a primitive basis, in particular spherical harmonics for the angular part and a finite-element discrete-variable-representation (FE-DVR) for the radial part[25,26]. The inner region consists of a linear combination of the primitive basis whose expansion coefficients are determined by a Hartree-Fock-like procedure[22]. We refer to Ref. 1, 22, 23 for details of our approach.

Numerical set-up

The primitive basis consists of spherical harmonics with quantum number l up to 10 and $|m|$ up to 1. The inner region of the basis consists of two finite elements, the first with 8 DVR functions within $r \in [0, 2)$ and the second element with 15 functions within $r \in [2, 15)$ (in atomic units). The outer region consists of 76 equally distributed elements containing 10 functions from $r = 15$ to $r < r_{\text{end}} = 600$. The total number of basis functions is then 21824. The convergence of the used basis has been carefully checked.

To avoid rescattering at the boundary, we use a complex absorbing potential (CAP) of the form[27,28]

$$V_{\text{CAP}} = -\mathrm{i}\left\{1 - \cos\left[\frac{\pi(|r| - r_{\text{CAP}})}{2(r_{\text{end}} - r_{\text{CAP}})}\right]\right\}, \quad r > r_{\text{CAP}}, \tag{25}$$

with $r_{\text{CAP}} = 500$ as the start of the CAP.

For the GAS division, we follow the work of Refs.[1,22] and use a complete-active-space (CAS) with single excitations out of the active space approximations, depicted schematically in Supplementary Figure 3. It contains a variable amount of orbitals ν and is denoted CAS*(2,ν). The more orbitals are included in the CAS, the more electronic correlations effect are taken into account and the better the accuracy of the simulation. In particular, ionization from excited states is then possible. In the context of quantum chemistry, CAS*(2,ν) would be denoted as multi-reference CIS. Regarding the selection of the orbitals in the CAS, we found good convergence with a CAS*(2,12), where orbitals up to the third shell with $m_{\max} = 1$ are included. This corresponds to approximately half a million Slater determinants. In the following, we denote the CAS*(2,12) as TDSE to simplify the notation.

The laser field utilized in the simulations consists of two parts stemming from the IR field and from the APT,

$$E(t) = E_{\text{IR}}(t) + E_{\text{XUV}}(\Delta t). \tag{26}$$

The IR field is described by a $\sin^2$ envelope,

$$E_{\text{IR}}(t) = \begin{cases} E_0^{\text{IR}} \sin(\omega t) \sin^2(\pi t/T_{\text{IR}}) & , t < T_{\text{IR}}, \\ 0 & , \text{else}. \end{cases} \tag{27}$$

The APT is modeled by a linear combination of phase-shifted XUV pulses with $\sin^4$-envelope:

$$E_{\text{XUV}}(\Delta t) = \sum_{n \in \{11,13,15,17\}} E_0^{\text{XUV}} b_n \sin(n\omega\Delta t + \beta_n) \sin(\pi\Delta t/T_{\text{XUV}})^4 \times E_{\text{XUV}}^{\sqcup}(\Delta t), \tag{28}$$

$$E_{\text{XUV}}^{\sqcup}(\Delta t) = \begin{cases} 1 & , \Delta t > T^{\text{XUV}} \wedge \Delta t > 0 \wedge t < T^{\text{IR}}, \\ 0 & , \text{else}. \end{cases} \tag{29}$$

The APT is shifted in units of IR cycles, t_{cycl},

$$\Delta t = t - \delta_{\mathrm{XUV}} t_{\mathrm{cycl}} - \frac{T_{\mathrm{IR}} - T_{\mathrm{XUV}}}{2}. \tag{30}$$

The last term in Supplementary Equation (30) is required to shift the maximum of the APT so that it coincides with the maximum of the IR pulse at $\delta_{\mathrm{XUV}} = 0$. The parameters are listed in Supplementary Table 1. The weights of the XUV components, b_n, are taken from the experiment. An example is shown in Supplementary Figure 4.

Simulation Results

Here we present the results of our simulations of XUV-initiated HHG. In order to illustrate the effect of the APT, we first compute the HHG spectrum from a simulation without the APT ($E_0^{\mathrm{XUV}} = 0$), and compare it to a simulation with the APT at zero delay ($\delta = 0$). Supplementary Figure 5 compares the two cases – tunneling-initiated and XUV-initiated HHG. The presence of the APT results in a pronounced increase of the intensities of harmonics 19 to 29, here up to eight orders of magnitude. Indeed, no trace of tunneling-initiated HHG was observed in the experiment.

The results from the XUV-IR delay scans are depicted in Supplementary Figure 6. We compare the calculated curves to the experimental data. Because delay zero, $\delta = 0$, cannot be retrieved from the experiment, the theory curves are shifted uniformly to match the first peak of harmonic 23. In order to obtain the intensity of a given harmonic from the simulations, we integrated over a spectral range of $\pm 0.1\omega$ around the central frequency of the harmonic. The agreement of the simulations and the experimental results is fair. We also observe significant anharmonicity of the oscillations, with a strong Fourier component at 4ω.

Supplementary Note 3: Preparatory measurements

Prior to the XUV-IR delay scan experiment, we performed a series of preparatory experiments. First, we recorded the spectrum of the xenon harmonics in the absence of helium gas. We optimized both the laser parameters and the focus position relative to the source gas cell for generating the brightest and least divergent harmonics. The local laser intensity in the cell was $8.7 \pm 1.9 \times 10^{13}\,\mathrm{W\,cm^{-2}}$. Second, we recorded the HHG spectrum of argon instead of helium atoms at the target gas cell (without xenon in the source gas cell). According to the measured cutoff energy, we evaluated the peak IR intensity at the target gas cell to be $7.8 \pm 1.9 \times 10^{13}\,\mathrm{W\,cm^{-2}}$. Third, we performed an absorption measurement of the xenon harmonics in helium by recording the spectrum while scanning the XUV-IR delay over a wide range of 300 fs so that we could observe the two extreme cases in which the IR pulse precedes the APT, and vice versa. According to the wide range absorption scan, we chose a narrow range (60 fs) for the high-resolution XUV-IR delay scan (see Supplementary Figure 8). We repeated the measurement for neon instead of helium atoms in the target gas cell (see Supplementary Figure 9), with a peak IR intensity of $5.2 \pm 1.9 \times 10^{13}\,\mathrm{W\,cm^{-2}}$ at the source gas cell and $5 \pm 2 \times 10^{13}\,\mathrm{W\,cm^{-2}}$ in the target gas cell.

Supplementary Note 4: Perturbative measurement experimental set-up

For the SH perturbative measurements, we used the laser system in a different configuration, with a pulse duration of 32 fs. We perturbed the interaction by adding the

second harmonic (SH) of the IR pulse (see Supplementary Figure 7). For that purpose, we generated the SH by propagating the IR beam through a barium boron oxide (BBO) crystal (thickness of 100 μm) followed by a half-wave plate, which matches the polarization of the pulses, before focusing them into the source gas cell. We control the relative sub-cycle temporal delay between the IR beam and the SH by a fused silica wedge pair with an accuracy of 80 as. The synchronization of the IR and the SH pulses is controlled by a series of calcite windows before the source gas cell, in conjunction with a fused silica window (thickness of 2 mm) between the gas cells, so that we can synchronize both pulses either solely in the source gas cell or in the target gas cell, by adding or reducing calcite thickness, respectively. We drilled a 3 mm diameter hole in the center of the fused silica window in order to allow propagation of the APT.

In addition to the preliminary experiments described above, we verified that the SH field is delayed sufficiently with respect to the IR pulse, so that the SH field does not affect HHG of xenon in the source gas cell. For this purpose, we performed an SH-IR delay scan while we recorded xenon HHG (no gas in the target gas cell). We could not detect even harmonics for a large range of SH intensities, which indicates that the SH perturbative HHG experiments are completely self-contained. We repeated these tests for the experiments in which we synchronized the SH and IR pulses in the source gas cell.

In the perturbative measurements in which we synchronized the SH and IR pulses in the target or the source gas cell, the SH-IR intensity ratio ε^2 was below 5% and 0.5%, respectively. For both measurements, the peak IR intensities in the source and target gas cells were $7.1 \pm 1.9 \times 10^{13}\,\mathrm{W\,cm^{-2}}$ and $7 \pm 3 \times 10^{13}\,\mathrm{W\,cm^{-2}}$, respectively. We typically scanned over 2-3 IR cycles for both the XUV-IR and SH-IR dimensions, with 60 as and 80 as accuracy, respectively. Since the measurement duration is relatively long (about two hours), it is prone to temporal and intensity drifts. In some cases, we observed systematic temporal drifts in the XUV-IR delay dimension, which we corrected.

Supplementary Note 5: Wide-range delay scans in helium and neon

Supplementary Figure 8 shows the intensity of harmonics 17 to 27 as a function of the XUV-IR delay over a delay range of 60 fs, in addition to the result we presented in Figure 2a in the main text. One can see that the envelope of the oscillations becomes narrower in time with ascending harmonic order. This response is expected since the generation of higher harmonics requires higher field intensities and the field intensity in the IR pulse gradually increases towards its temporal center. All harmonics oscillate with high contrast, motivating the use of Coulomb-corrected three-step model (see Supplementary Note 1). As described above, we repeated the experiment with neon instead of helium in the target gas cell. Supplementary Figure 9 shows the intensity oscillations of harmonics 15-19 as a function of XUV-IR delay. Here we generated 3 harmonics above the neon ionization threshold since we used a lower IR peak intensity. Using the same IR peak intensity as in the helium experiment, the lower ionization threshold of neon already allows tunneling-initiated HHG from its ground state, which adds a significant background.

Supplementary Note 6: Data acquisition and analysis

All results from the experiments are based on spectroscopic measurements. For every XUV-IR delay, we measured the average spectrum over 100 IR pulses. Next, for each harmonic we set a specific region of interest (ROI) and integrated over it (see Supplementary Figure 10). We used the ROI mask to extract the intensity of each harmonic as a function of XUV-IR delay. The constant background for each harmonic was determined according

to the measured intensity at large XUV-IR delays where the APT and the IR pulse do not overlap temporally, and it was subsequently subtracted. We Fourier transformed the data within a window of 5 oscillation cycles and varied the offset delay of the window by ± 2 oscillation cycles in order to obtain statistics of the signals. We extracted the average values of Fourier amplitudes and phases which form the basis of the reconstruction procedure. In this approach, the error bars are given by the standard deviations of the amplitudes and phases (see Figure 7 in the Methods section of the main text), and hence indicate how robust the signal is to the choice of the integration window.

In the SH perturbative measurements, we raster scanned over both the XUV-IR and SH-IR delays, and recorded the spectrum for every delay point. For each harmonic (including the even harmonics in these experiments), we followed the same steps described above for integration and background subtraction. Accordingly, we created a two-dimensional intensity map for each harmonic, as a function of both delays. Next, we summed over the dimensions cycle wise, forming an integrated map (typically we scan over 3 oscillation cycles for each dimension). We followed the same steps in order to extract the optimal delays for the XUV-IR dimension as described above. For the SH-IR phases, we integrated over all the XUV-IR delays, fit a sinusoidal function to the oscillating harmonics intensity and extracted the associated SH-IR phase. The errors for the XUV-IR delay scans are dominated by the position accuracy of the piezo stage, which moves the XUV mirror and hence, control the XUV-IR delay. The errors in the SH-IR phases are calculated according to the standard deviation of the sinusoidal fit.

SUPPLEMENTARY REFERENCES

[1] Larsson, H. R., Bauch, S., Sørensen, L. K. & Bonitz, M. Correlation effects in strong-field ionization of heteronuclear diatomic molecules. *Phys. Rev. A* **93**, 013426 (2016).

[2] Corkum, P. B. Plasma perspective on strong field multiphoton ionization. *Phys. Rev. Lett.* **71**, 1994–1997 (1993).

[3] Lewenstein, M., Balcou, P., Ivanov, M. Y., L'Huillier, A. & Corkum, P. B. Theory of high-harmonic generation by low-frequency laser fields. *Phys. Rev. A* **49**, 2117–2132 (1994).

[4] Figueira de Morisson Faria, C. & Salières, P. High-order harmonic generation with a strong laser field and an attosecond-pulse train: The Dirac-Delta comb and monochromatic limits. *Laser Physics* **17**, 390–400 (2007).

[5] Leeuwenburgh, J., Cooper, B., Averbukh, V., Marangos, J. P. & Ivanov, M. Y. High-order harmonic generation spectroscopy of correlation-driven electron hole dynamics. *Phys. Rev. Lett.* **111**, 123002 (2013).

[6] Hostetter, J. A., Tate, J. L., Schafer, K. J. & Gaarde, M. B. Semiclassical approaches to below-threshold harmonics. *Phys. Rev. A* **82**, 023401 (2010).

[7] He, F., Ruiz, C., Becker, A. & Thumm, U. Attosecond probing of instantaneous ac Stark shifts in helium atoms. *J. Phys. B: At. Mol. Opt. Phys.* **44**, 211001 (2011).

[8] Singh, K. P. & Rost, J. M. Global control of attosecond photoionization of atoms through XUV dispersion. *Phys. Rev. A* **91**, 013415 (2015).

[9] Schafer, K. J., Gaarde, M. B., Heinrich, A., Biegert, J. & Keller, U. Strong field quantum path control using attosecond pulse trains. *Phys. Rev. Lett.* **92**, 023003 (2004).

[10] Gaarde, M. B., Schafer, K. J., Heinrich, A., Biegert, J. & Keller, U. Large enhancement of macroscopic yield in attosecond pulse train-assisted harmonic generation. *Phys. Rev. A* **72**, 013411 (2005).

[11] Gademann, G. *et al.* Attosecond control of electron-ion recollision in high harmonic generation. *New J. Phys.* **13**, 033002 (2011).

[12] Fehlberg, E. Classical Runge–Kutta formulas of fourth and lower order with stepsize control and their use for heat problems. *Computing* **6**, 61–71 (1970).

[13] Chen, S., Ruiz, C. & Becker, A. Double ionization of helium by intense near-infrared and VUV laser pulses. *Phys. Rev. A* **82**, 033426 (2010).

[14] Skruszewicz, S. *et al.* Two-color strong-field photoelectron spectroscopy and the phase of the phase. *Phys. Rev. Lett.* **115**, 043001 (2015).

[15] Simpson, E. R. *et al.* Polarisation response of delay dependent absorption modulation in strong field dressed helium atoms probed near threshold. *New J. Phys.* **18**, 083032 (2016).

[16] Gaarde, M. B., Tate, J. L. & Schafer, K. J. Macroscopic aspects of attosecond pulse generation. *J. Phys. B: At. Mol. Opt. Phys.* **41**, 132001 (2008).

[17] McCurdy, C. W., Baertschy, M. & Rescigno, T. N. Solving the three-body coulomb breakup problem using exterior complex scaling. *J. Phys. B: At. Mol. Opt. Phys.* **37**, R137–R187 (2004).

[18] Hochstuhl, D., Hinz, C. & Bonitz, M. Time-dependent multiconfiguration methods for the numerical simulation of photoionization processes of many-electron atoms. *Eur. Phys. J. Special Topics* **223**, 177–336 (2014).

[19] Han, Y.-C. & Madsen, L. B. Comparison between length and velocity gauges in quantum simulations of high-order harmonic generation. *Phys. Rev. A* **81**, 063430 (2010).

[20] Bandrauk, A. D., Chelkowski, S., Diestler, D. J., Manz, J. & Yuan, K.-J. Quantum simulation of high-order harmonic spectra of the hydrogen atom. *Phys. Rev. A* **79**, 023403 (2009).

[21] Sørensen, L. K., Bauch, S. & Madsen, L. B. The integral screened configuration interaction method (2016). arXiv:1609.07757.

[22] Bauch, S., Sørensen, L. K. & Madsen, L. B. Time-dependent generalized-active-space configuration-interaction approach to photoionization dynamics of atoms and molecules. *Phys. Rev. A* **90**, 062508 (2014).

[23] Hochstuhl, D. & Bonitz, M. Time-dependent restricted-active-space configuration-interaction method for the photoionization of many-electron atoms. *Phys. Rev. A* **86**, 053424 (2012).

[24] Olsen, J., Roos, B. O., Jørgensen, P. & Jensen, H. J. A. Determinant based configuration interaction algorithms for complete and restricted configuration interaction spaces. *J. Chem. Phys.* **89**, 2185–2192 (1988).

[25] Balzer, K., Bauch, S. & Bonitz, M. Efficient grid-based method in nonequilibrium Green's function calculations: Application to model atoms and molecules. *Phys. Rev. A* **81**, 022510 (2010).

[26] Rescigno, T. & McCurdy, C. Numerical grid methods for quantum-mechanical scattering problems. *Phys. Rev. A* **62**, 032706 (2000).

[27] Zanghellini, J., Jungreuthmayer, C. & Brabec, T. Plasmon signatures in high harmonic generation. *J. Phys. B: At. Mol. Opt. Phys.* **39**, 709 (2006).

[28] Miyagi, H. & Madsen, L. B. Time-dependent restricted-active-space self-consistent-field theory for laser-driven many-electron dynamics. *Phys. Rev. A* **87**, 062511 (2013).
